# Accelerometer measured daily physical activity and sedentary pursuits–comparison between two models of the Actigraph and the importance of data reduction

**DOI:** 10.1186/1756-0500-6-439

**Published:** 2013-10-31

**Authors:** Tina Tanha, Åsa Tornberg, Magnus Dencker, Per Wollmer

**Affiliations:** 1 Department of Clinical Sciences, Unit of Clinical Physiology and Nuclear Medicine, Skåne University Hospital, Lund University, Malmö, Sweden; 2 Department of Cardiology, Heart Failure and Valvular Disease, Skåne University Hospital, Lund University, Malmö, Sweden; 3 Department of Health Sciences, Physiotherapy, Lund University, Malmö, Sweden; 4 Department of Clinical Sciences, Genetic & Molecular Epidemiology Unit, Lund University Diabetes Center, Skåne University Hospital, Malmö, Sweden

**Keywords:** Accelerometers, Accelerometry, Validation study, Actigraph

## Abstract

**Background:**

Very few validation studies have been performed between different generations of the commonly used Actigraph accelerometers. We compared daily physical activity data generated from the old generation Actigraph model 7164 with the new generation Actigraph GT1M accelerometer in 15 young females for eight consecutive days. We also investigated if different wear time thresholds had any impact on the findings. Minutes per day of moderate and vigorous physical activity (MVPA), vigorous physical activity (VPA) and very vigorous physical activity (VVPA) were calculated. Moreover, minutes of sedentary pursuits per day were calculated.

**Findings:**

There were significant (P < 0.05) differences between the Actigraph 7164 and the GT1M concerning MVPA (61 ± 21vs. 56 ± 23 min/day), VPA (12 ± 8 vs. 9 ± 3 min/day) and VVPA (3.2 ± 3.0 vs. 0.3 ± 1.1 min/day). The different wear time thresholds had little impact on minutes per day in different intensities. Median minutes of sedentary pursuits per day ranged from 159 to 438 minutes depending on which wear time threshold was used (i.e. 10, 30 or 60 minutes), whereas very small differences were observed between the two different models.

**Conclusions:**

Data from the old generation Actigraph 7164 and the new generation Actigraph GT1M accelerometers differ, where the Actigraph GT1M generates lower minutes spent in free living physical activity. Median minutes of sedentary pursuits per day are highly dependent on which wear time threshold that is used, and not by accelerometer model.

## Findings

Accelerometers are widely used for assessment of physical activity in childhood to old age [1-3]. They provide objective and detailed information on various aspects of physical activity [4-6]. The Actigraph is probably the most validated accelerometer on the market [6]. Validation studies have established appropriate cut-off points corresponding to different intensity levels for the old generation Actigraph 7164 [7,8]. Newer generations of the Actigraph have been introduced, and since accelerometers are increasingly used in research regarding physical activity and data output from older models are compared and used with newer models, it appears important to evaluate potential differences between different accelerometer generations. After the accelerometer data are collected there are different ways of analysing the data concerning wear time (i. e. if the accelerometer has been worn or not), unfortunately without consensus on how.

This study therefore aims to evaluate:

1. Possible differences between two types of accelerometers, an old generation Actigraph 7164 and a new generation Actigraph GT1M in daily physical activity measurements, including minutes of sedentary pursuits per day.

2. The impact of different wear time thresholds on activity measurements.

## Methods

A total of 15 females aged 18-32 years were investigated. The participants were soccer players from a local soccer team. Weight and height were measured, and BMI was calculated. The study was approved by the Regional ethical review board at Lund, and performed according to the Helsinki Declaration. Written consent was obtained from the participants. Both accelerometers (Actigraph 7164 and Actigraph GT1M, Actigraph inc., Pensacola, FL) were worn side-by-side around the waist for eight consecutive days. The participants were instructed to use the accelerometer during waking hours and only remove it during activities that could damage the accelerometer (water based activities). A recording epoch of 60 seconds was selected for this study. Eight hours of valid recording was considered an acceptable day [2].

Accelerometer data were assessed in ActiLife 5.10.0 (Actigraph inc., Pensacola, FL). Minutes per day of moderate and vigorous physical activity (MVPA), vigorous physical activity (VPA) and very vigorous physical activity (VVPA) were calculated. Moreover, minutes of sedentary pursuits per day were calculated. Sedentary pursuits were defined as <100 counts per min (cpm) [9], MVPA as >1952, VPA as >5725 and VVPA as > 9499 cpm [7,8]. Three different wear time thresholds were applied with the software from the manufacturer with threshold values: 10, 30 and 60 minutes (defined as 10, 30 or 60 minutes of consecutive zeros in the accelerometer data). Thus, if 10 minutes of wear time threshold was used, it meant that all data during the 10 minutes of consecutive zeros were deleted. We also performed an additional analysis with allowance of 2 minutes of sporadic activity counts.

Statistica 10 (StatSoft Inc, Tulsa, OK, USA) was used for all statistical analyses. Descriptive data are presented as mean ± SD and accelerometer data as median ± IQR (interquartilrange). Differences between medians for the Actigraph 7164 and the Actigraph GT1M were tested with the Wilcoxon matched pairs test. A ratio between data from Actigraph 7164 and the Actigraph GT1M was calculated.

## Results

The participants were 23.7 ± 4 years (mean ± SD), body mass 65 ± 6 kg, height 171 ± 4 cm and BMI 22 ± 1 kg/m^2^. All participants had eight acceptable recording days [2]. Consistently lower median minutes per day spent in all different physical activity levels were found for Actigraph GT1M compared to Actigraph 7164, where the relative difference was higher at higher intensities (i. e. VPA and VVPA). Median minutes of sedentary pursuits per day ranged from 159 to 438 minutes depending on which wear time threshold was used (i.e. 10, 30 or 60 minutes), whereas very small differences were observed between the two different models. Table 1 summarises the accelerometer findings. Incorporation of 2 minute segments of sporadic activity during the wear time threshold did not have any major impact on the results with the exception that the differences for VPA failed to reach statistical significance (data not shown).

**Table 1:** Comparison between the old Actigraph model 7164 and the new Actigraph GT1M depending on wear time threshold (i.e. 10, 30 or 60 minutes wear time), values are median ± IQR (interquartile range)

**10 min wear time**				
	Mod 7164	GT1M	P-value	7164/ GT1M
Sedentary (<100 cpm)	184 ± 50	159 ± 36	0.12	1.16
MVPA (>1952 cpm)	61 ± 20	56 ± 23	0.01	1.09
VPA (>5725 cpm)	12 ± 8	9 ± 3	0.002	1.33
VVPA (>9499 cpm)	3.2 ± 3.0	0.3 ± 1.1	<0.001	10.67
**30 min wear time**				
	Mod 7164	GT1M	P-value	7164/ GT1M
Sedentary (<100 cpm)	337 ± 76	331 ± 64	1.0	1.02
MVPA (>1952 cpm)	61 ± 21	56 ± 23	0.01	1.09
VPA (>5725 cpm)	12 ± 8	9 ± 3	0.002	1.33
VVPA (>9499 cpm)	3.2 ± 3.0	0.4 ± 1.1	<0.001	8.00
**60 min wear time**				
	Mod 7164	GT1M	P-value	7164/ GT1M
Sedentary(<100 cpm)	429 ± 64	438 ± 53	0.01	0.98
MVPA (>1952 cpm)	61 ± 21	56 ± 23	0.01	1.09
VPA (>5725 cpm)	12 ± 8	9 ± 3	0.002	1.33
VVPA (>9499 cpm)	3.2 ± 3.0	0.4 ± 1.1	<0.001	8.00

Moderate and vigorous physical activity (MVPA), vigorous physical activity (VPA) and very vigorous physical activity (VVPA). The ratio between median data output of 7164 and GT1M is also expressed.

## Discussion

This was a comparison between old generation Actigraph model 7164 and new generation Actigraph GT1M in free-living physical activity. The new generation Actigraph GT1M showed consistently lower median minutes per day for the investigated intensities, whereas small differences were observed for time in sedentary pursuits. Different wear time thresholds had no effect on minutes in different physical activity intensities. In contrast, time in sedentary pursuits was dependent on which wear time threshold that was used.

There are only a few studies in this field [10,11], and the findings are diverging. has previously compared the GT1M and 7164 during treadmill testing and found significant differences ranging from 10 to 23% between the models [12]. Corder et al. also compared the Actigraph model 7164 with the Actigraph GT1M [9]. Free-living daily activity was measured with both models for seven days, however in contrast to this study their population consisted of 15-16 year old teenagers. Their results indicated no significant differences between the different models for time spent in moderate or vigorous physical activity [10]. However, time performing light activity was 9% lower with the Actigraph GT1M. In contrast to Corder et al. we observed daily physical activity values for all intensities [10]. One suggestion to why data output between the Actigraph models differ more at low intensities than high intensity is that accelerometer counts tend to reach a plateau, which has previously been described [13,14]. Moreover, John et al. compared the Actigraph (GT1M and 7164) during a treadmill test in ten endurance trained men (ages 23 ± 3) and concluded no significant differences between the different models [11]. In contrast, the results of this study suggest differences in Accelerometer output between Actigraph 7164 and GT1M. One feasible explanation can be the technical differences between Actigraph 7164 and GT1M regarding band pass filtering and sampling frequency [14,15]. The new Accelerometers have more advanced electronic properties, for example the GT1M digitizes the accelerometer output by a 12-bit analog to digital converter at 30 Hertz in comparison to 7164 that digitize by 8-bit analog to digital converter at 10 Hertz [16]. These differences may result in the differences found in data output between the two models. Regardless of the reason we conclude that previous data from validation and field-based studies from old generation Actigraph models may not be interchangeable. It is therefore important to generate new validation studies with the new generation accelerometers since the present validation studies and cut-off points are mainly carried out with the old generation accelerometers [6-8]. The diverging findings between studies also indicate need for large-scale comprehensive validation studies. The differences between the different Actigraph models seem to be between 7164 and GT1M, as two recent studies have not shown any differences between the GT1M and the GT3X [17,18] and during uniaxial setting [19]. Ried-Larsen et al compared 7164 with GT3X in a free living setting with a population of 20 (mean age 37.8) and found significant differences in data output between the monitors [17]. They did not compare 7164 with GT1M in a free living setting, which differs from our study. However, there was a significant difference between 7164 and GT1M in the mechanical setup.

There has in recent years also been an increased interest in health-related implications specifically associated with sedentary pursuits [9,20-22]. A small difference (mean output ratio 7164/GT1M = 0.95) was seen when 60 minute wear time threshold was used in median minutes of sedentary pursuit between the models. No significant differences were observed when 10 or 30 minute wear time thresholds were used. It is difficult to say why no differences occurred between the models when measuring <100 cpm in contrast to activities >1952 cpm, but one line of reasoning may be that the absolute values <100 are so small that it requires very large differences to obtain significance. However the analyses of different wear time thresholds showed that there was almost a threefold difference in minutes of sedentary pursuits per day between the lowest threshold value 10 min and the highest threshold value 60 min. The reasonable explanation is that selection of a higher wear time threshold will presumably result in time that the accelerometer is not worn being misinterpreted as sedentary pursuits. There is, however, no consensus whether to delete missing data or not. Many early studies did not report deletion of missing data and probably did not do it. Several investigators have used the practice of deleting missing data defined as 10 minutes of zeros [23-26], some have defined missing data as 20 minutes of zeros [27], and some have used one hour with allowance of sporadic episodes of low counts [28]. A completely different approach of replacing missing data with various type estimates has also been suggested [29], which is somewhat questionable since it makes the assumption that episodes that the monitor is not worn represent average physical activity. This assumption is highly dependent on whether the monitor was simply forgotten, or taken off because of a certain activity such as for example swimming. There are some studies [30-32] that have examined correlations of different wear time thresholds and agree that time spent in sedentary pursuit is effected by which type of wear time threshold is used. However, wear time threshold has no or small influence on MVPA. Instead, it seems that increasing the wear time threshold decreases the average activity count per minute. There are multiple algorithms suggested to improve the quality of accelerometer data, one being allowance of 2 minute segments during the wear time threshold [28,33]. This analysis was performed in this study and did not have any major impact on the results.

Limitation of this study includes the low number of participants, which may influence power. This was, however, a pilot study. Additional limitations were that only females were investigated and that we did not have access to a gold standard (i.e. indirect calorimetry).

## Conclusion

The older generation Actigraph 7164 consistently generates lower median minutes spent in MVPA, VPA and VVPA compared to Actigraph GT1M in a free living physical activity setting. Accelerometer model does not influence median minutes of sedentary pursuit, instead differences are dependent on which wear time threshold is used.

## Competing interests

The authors declare that they have no competing interests.

## Authors’ contributions

All authors made substantial contributions to conception and design. ÅT and TT made substantial contributions to acquisition of data. TT, ÅT and MD made substantial contributions to analysis and interpretation of data. TT, MD and PW made substantial contributions to drafting the article. All authors made substantial contributions to revising it critically for important intellectual content and gave final approval of the version to be published.
